# Burst intensification by singularity emitting radiation in multi-stream flows

**DOI:** 10.1038/s41598-017-17498-5

**Published:** 2017-12-21

**Authors:** A. S. Pirozhkov, T. Zh. Esirkepov, T. A. Pikuz, A. Ya. Faenov, K. Ogura, Y. Hayashi, H. Kotaki, E. N. Ragozin, D. Neely, H. Kiriyama, J. K. Koga, Y. Fukuda, A. Sagisaka, M. Nishikino, T. Imazono, N. Hasegawa, T. Kawachi, P. R. Bolton, H. Daido, Y. Kato, K. Kondo, S. V. Bulanov, M. Kando

**Affiliations:** 1 Present Address: Kansai Photon Science Institute, National Institutes for Quantum and Radiological Science and Technology, 8-1-7 Umemidai, Kizugawa-city, Kyoto 619-0215 Japan; 2 Graduate School of Engineering, Osaka University, 2-1 Yamadaoka, Suita, Osaka 565-0871 Japan; 3 Joint Institute for High Temperatures of the Russian Academy of Sciences, Izhorskaja Street 13/19, Moscow, 127412 Russia; 4 Open and Transdisciplinary Research Initiatives, Osaka University, Suita, Osaka 565-0871 Japan; 5 P. N. Lebedev Physical Institute of the Russian Academy of Sciences, Leninsky Prospekt 53, Moscow, 119991 Russia; 6 Moscow Institute of Physics and Technology (State University), Institutskii pereulok 9, Dolgoprudnyi, Moscow Region 141700 Russia; 7 Central Laser Facility, Rutherford Appleton Laboratory, STFC, Chilton, Didcot, Oxon OX11 0QX UK; 8 Department of Physics, SUPA, University of Strathclyde, Glasgow, G4 0NG UK; 9 Naraha Remote Technology Development Center, Japan Atomic Energy Agency, Naraha-machi, Fukushima, 979-0513 Japan; 10 The Graduate School for the Creation of New Photonics Industries, 1955-1 Kurematsu-cho, Nishiku-Hamamatsu, Shizuoka 431-1202 Japan; 11 A. M. Prokhorov Institute of General Physics of the Russian Academy of Sciences, Vavilov Street 38, Moscow, 119991 Russia; 12 Institute of Physics of the Czech Academy of Sciences, v.v.i. (FZU), ELI-Beamlines Project, Na Slovance 1999/2, 182 21 Prague, Czech Republic; 13 Present Address: Chair of Experimental Physics and Medical Physics, Faculty of Physics, Ludwig-Maximilians- Universität München, Am Coulombwall 1, D-85748 Garching b., München Germany

## Abstract

Burst Intensification by Singularity Emitting Radiation (BISER) is proposed. Singularities in multi-stream flows of emitting media cause constructive interference of emitted travelling waves, forming extremely localized sources of bright coherent emission. Here we for the first time demonstrate this extreme localization of BISER by direct observation of nano-scale coherent x-ray sources in a laser plasma. The energy emitted into the spectral range from 60 to 100 eV is up to ~100 nJ, corresponding to ~10^10^ photons. Simulations reveal that these sources emit trains of attosecond x-ray pulses. Our findings establish a new class of bright laboratory sources of electromagnetic radiation. Furthermore, being applicable to travelling waves of any nature (e.g. electromagnetic, gravitational or acoustic), BISER provides a novel framework for creating new emitters and for interpreting observations in many fields of science.

## Introduction

Bright, compact sources of ultra-short x-ray pulses is a hot research topic^1–7^ and are demanded in many applications^8,9^. They are exclusively based on laser-matter interactions. In our recent experiments^10,11^, we have observed bright coherent soft x-rays with comb-like spectra emitted from low-density relativistic laser plasmas. The properties of this emission cannot be explained by previously known mechanisms such as Thomson scattering^2,4^, betatron radiation^3,5^ and atomic harmonics from the recollision process^1,12^. The observed x-ray photon number is orders of magnitude greater than the most favourable estimates for Thomson scattering. The emission consists of harmonics of optical frequency, unlike betatron radiation. Both linearly and circularly polarized lasers produce odd and even harmonics orders, in contrast to the recollision process^12^. In order to explain our results we have proposed a new mechanism^10,11^, where this emission originates from relativistic electron density singularities, i.e. point-like sources.

Here, for the first time we confirm this cornerstone prediction demonstrating unprecedented nanoscale sources of bright coherent soft x-rays in laser plasma. This establishes a new class of compact x-ray sources of ultrashort duration, demanded in numerous applications. In addition, the analysis of the emission mechanism allows us to generalise it to travelling waves of any nature, such as electromagnetic, gravitational or acoustic types, and propose the concept of Burst Intensification by Singularity Emitting Radiation (BISER).

## The BISER concept

**Figure 1:**
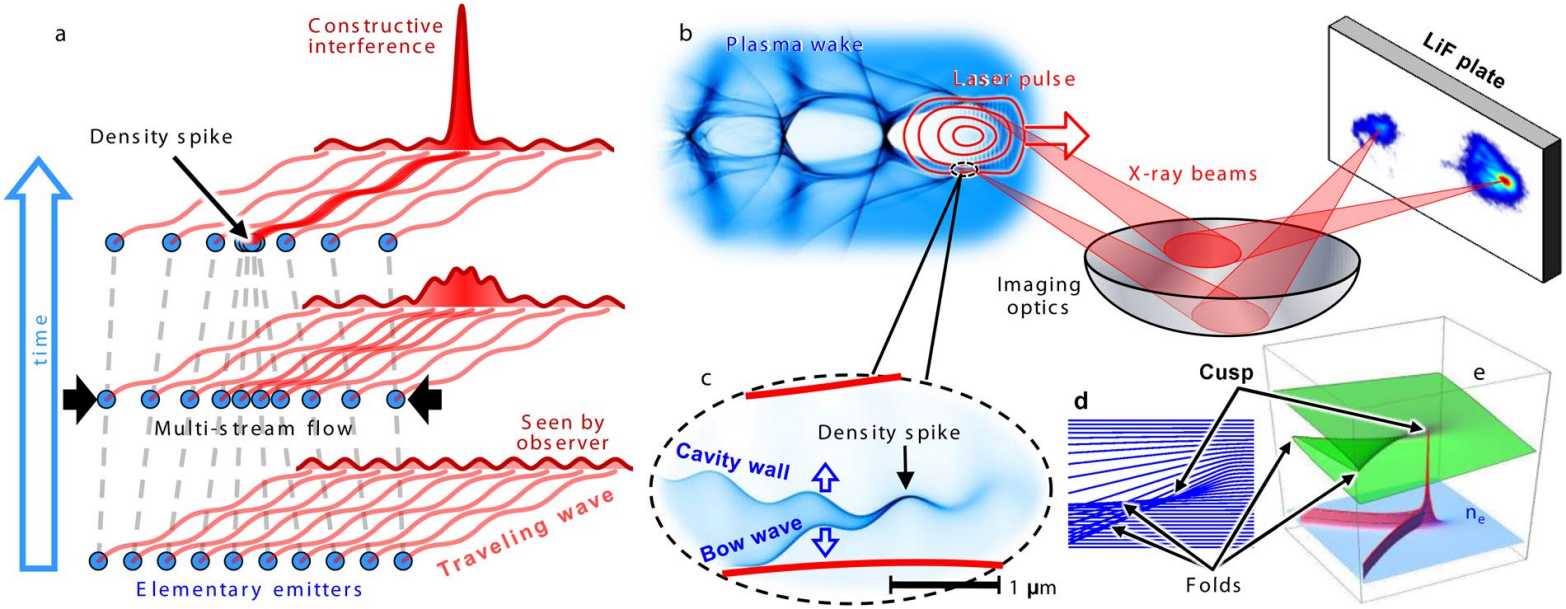
Formation of a singularity (density spike) and corresponding burst intensification of emitted radiation. (**a**) Multi-stream converging flow consisting of elementary emitters eventually forms a density spike capable of generating a burst of coherent emission. (**b**) In the experiment, the near-infrared laser pulse propagates in the plasma creating density spikes, which produce x-rays that are collected by imaging optics and recorded by a LiF plate. (**c**) A density spike appears in a multi-stream flow of electrons pushed aside by the laser field (PIC simulation). (**d**, **e**), A multi-stream flow model, where the electron transverse displacement forms regions of high concentration, corresponding to density catastrophes–point-like cusp and folds.

Multi-stream flows^13^ ubiquitously occur in media (such as with shock waves^14,15^ and jets^16^ in astrophysical and laboratory plasmas^14,17^). Converging flows can lead to catastrophic changes of the medium characteristics, e.g. formation of fast moving singularities such as long-lived density spikes, which are robust with respect to perturbations. Their existence, universality and structural stability are explained by catastrophe theory^18,19^. In various physical media the elementary components can emit travelling waves. We propose that, in such a medium, a high concentration and synchronism in the singularities bring constructive interference of the emitted travelling waves, creating Burst Intensification by Singularity Emitting Radiation (BISER), Fig. 1a. This emission originates from regions with the size well below the emitted wavelength, Λ_E_. It is both *spatially* and *temporally* coherent, if the emitted wave phase is a continuous function of the emitter coordinates and/or momenta. This condition is satisfied, e.g., when the elementary emitters are driven (excited) by an external field, whose spatial scale is greater than Λ_E_. The resulting emission intensity scales quadratically^20^ with the number of elementary emitters in the singularity, in contrast to the linear scaling of the intensity of incoherent emission from the background. If the singularity moves with a relativistic speed corresponding to the Lorentz factor *γ*, its emission is confined in a narrow angle of the order of 1/*γ* in the motion direction, shifts to shorter wavelengths^21^, λ_E_ ~ Λ_E_/2*γ*, and becomes at least *γ* ^4^ times more intense^20,22^. It is essential that the singularity consists of elementary emitters continuously flowing through it. In this respect it fundamentally differs from the case of a compact bunch^23^, which consists of the same elementary emitters. Further, in sharp contrast to a particle bunch, the singularity is determined by the multi-stream flow and has a non-local nature. In both cases only the waves emitted from the location of the highest density interfere constructively.

Laser plasma is an ideal medium for realization and study of the BISER effect in the laboratory, under controllable conditions. For the sake of clarity, here we describe how BISER explains previous experiments^10,11^, where the medium is a laser plasma and the travelling waves are electromagnetic ones. An ultra-intense laser quickly ionizes matter creating a relativistic plasma^24^, where multi-stream flows and singularities emerge, e.g., at the longitudinal^25^ and transverse breaking^26^ of nonlinear Langmuir waves^27,28^. When a tightly focused laser pulse propagates in low-density plasma, Fig. 1b, it pushes electrons forming a cavity in the electron density and a bow wave^29^, Fig. 1c. The resulting transverse displacement of electrons, Fig. 1d, produces density singularities of different dimensionality, Fig. 1e, seen as a density spike and sharp boundaries of the cavity wall and bow wave. Due to the modulations imposed by the laser with the scale of the laser wavelength, Fig. 1c, the density spike oscillates, emitting high-frequency electromagnetic radiation^10,11^ in a cone around its velocity vector, analogously to a relativistic oscillating electric charge. Remarkably, the density singularities are robust with respect to laser-imposed modulation, because they correspond to structurally stable catastrophes^18,19^. Sharp outlines of the cavity wall and bow wave, Fig. 1c, correspond to fold catastrophes. The density spike corresponds to a higher order cusp catastrophe at the joint of two folds. The cusp is located on a line encircling the laser pulse, in contrast to the transverse wave breaking^26^ which produces singularities at the end of the first period of the Langmuir wave. A linearly polarized laser pulse strongly pushes electrons along the polarization axis, creating higher concentration and stronger modulation of electrons at the two opposite points on the cusp line. Since the radiation intensity at constructive interference quadratically depends on the electron number, these two points represent the strongest emitters.

### Experiment

We realised BISER in the interaction between a multi-terawatt femtosecond laser, Supplementary Figure 1, with the estimated peak irradiance ranging from *I*_0_ ≈ 6 × 10^19^ to 2 × 10^20^ W/cm^2^ and a supersonic helium jet with the electron density from *n*_e_ ≈ 1.4 × 10^19^ to 6 × 10^19^ cm^−3^, using high spatial resolution soft x-ray diagnostics, Supplementary Figures 2–5. We sequentially used two detectors, a Spectrograph equipped with a back-illuminated x-ray CCD (Supplementary Figure 2) and an Imager equipped with a LiF plate (Supplementary Figure 3). The acceptance angle of these detectors, ±5° cone around the central angle of 13° with respect to the laser propagation direction, was determined by a normal-incidence multilayer mirror (“imaging optics” in Fig. 1b).

Using the Spectrograph’s ability of a readout on every laser shot, we performed a survey of the laser-plasma interaction varying the laser pulse energy, gas jet pressure, and position of the laser focus inside the gas jet. Under a wide range of experimental conditions stated above, we observed coherent soft x-ray radiation, always originating from point-like emitters. The brightness of these emitters exceeded that of the background laser plasma by many orders of magnitude. At the optimum conditions, this radiation was observed in ~90% of the shots. The brightest emission corresponded to two emitting regions seen as bright spots in Supplementary Figure 2e, in the plane perpendicular to the observation direction. The distance between the emitting regions was from 10 to 20 μm. They were situated along the laser polarization plane, as predicted by the theory.

**Figure 2:**
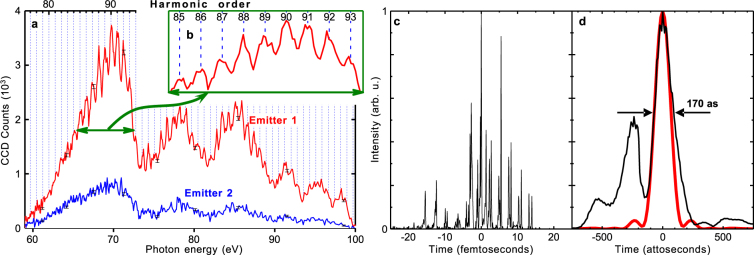
Properties of singular emitters. (**a**) Spectra of two point-like emitters obtained in a single shot (experiment). (**b**) Inset shows the harmonic structure in more detail. (**c**) Temporal structure of the harmonic pulse exhibiting an attosecond pulse train (PIC simulation). (**d**) The strongest attosecond pulse in the train (c) with the duration of 170 as. The red line shows the bandwidth-limited 150 attosecond pulse estimated from the experimental spectrum in the 60 to 90 eV range, panel (a).

The spectra of the point-like emitters were comprised of high-order harmonics of the optical frequency, Fig. 2a,b. The harmonics were not always resolved, which might be connected either with a single emission spike in the time domain^8^ or defocusing, because even a few-tens µm shift of the source position in the observation direction would result in the spectral image blurring and corresponding lower spectral resolution. Two different types of modulations were seen in the harmonic spectra, Fig. 2. The modulations with the period ~1 eV were the optical harmonic comb associated with the attosecond pulse train, Fig. 2c, as shown below in the Simulation section. The modulations with a larger scale (from ~5 to ~10 eV) were due to the spectrograph throughput, Supplementary Figure 4a.

The angular distribution of the soft x-ray emission was measured using the Spectrograph in the strongly defocused mode, when the spherical mirror imaged a plane situated a few mm downstream from the source, rather than the source itself. In this case, the pixel position on the CCD corresponded to the observation angle, rather than position in the source plane (object space). The precise angular scale calibration was performed using the shadow of the 150 µm period parallel support mesh of the transmission grating, Supplementary Figure 2b. In the laser polarization plane, the off-axis radiation typically extended to 10–13° and in some shots exceeded 18°, the outer mirror’s edge. The full divergence estimated from these data was 20–30°, consistent with the small source size. In the perpendicular plane, the typical divergence was 10°.

The pulse energy and absolute number of photons in the soft x-ray spectra were estimated using the calculated mirror reflectivity which agreed with measurements at several wavelengths^30^, measured filters transmission^31^, calculated transmission grating efficiency^32,33^, and CCD efficiency and gain provided by the manufacturer. In this way the Spectrograph throughput, Supplementary Figure 4a, was obtained. Supplementary Figure 5 gives the spectrum in absolute units of the stronger emitter of the shot shown in Fig. 2a and Supplementary Figure 2. The energy emitted into the acceptance angle and observable spectral range from 60 to 100 eV was up to ~100 nJ, corresponding to ~10^10^ photons.

**Figure 3:**
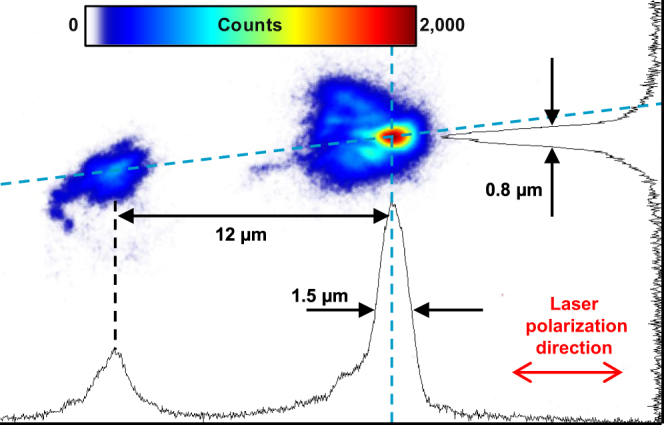
Singular emitters revealed in the experiment. Single-shot image produced on the LiF plate by photons with the energy from 60 to 100 eV. The solid lines show lineouts along the light dashed lines.

Since the CCD pixel size in the Spectrograph did not enable achieving sufficiently high spatial resolution, we set experimental parameters corresponding to the strongest soft x-ray emission and used the Imager for obtaining the high-resolution images of the emitters, Fig. 3. The stronger emitter is shown in Supplementary Figure 3b. The determination of its size, apparently 1.5 μm by 0.8 μm, was limited by the imaging system imperfections. However, the prominent fringes seen in the lineout, Supplementary Figure 3b, suggested that the transverse size was substantially smaller. Indeed, physical optics propagation modelling provided a conservative estimate of the transverse source size to be not greater than 100 nm; for larger sizes the modelling showed much fainter blurred fringes. For comparison, the initial laser spot size was about 10 μm, Supplementary Figure 1b, while an incoherent plasma emission was observed from a 0.5 mm region, Supplementary Figure 2c.

The radiation was spatially and temporally coherent, as evidenced by the spatial and spectral fringes, Fig. 3 and Fig. 2b, respectively. We emphasize that the propagation effect (van Cittert-Zernike theorem)^34^ cannot explain the spatial coherence of our point-like source. The image in Fig. 3 was formed by a focusing mirror situated at the distance *r* = 286 mm from the source. The fine fringes seen in Fig. 3 and lineout of Supplementary Figure 3b indicate that the whole beam reflected by the mirror was spatially coherent. The measurements of the soft x-ray angular distribution discussed above showed that the illuminated mirror area size ranged from *D* = 20 to 48 mm (the full mirror width). For the central wavelength of λ_E_ = 15 nm, the propagation coherence effect becomes applicable (the far field condition) only at the distance *r* ~ *D* ^2^/λ_E_ > 20 km, many orders of magnitude greater than the distance in the experiment. Thus the fringes appeared in the image due to the source coherence, not due to the propagation. This fundamentally differs from a typical phase contrast experiment with an incoherent source, where only a local coherence is sufficient, and the coherence width is usually *D* ~ 0.01 to 0.1 mm while *r* ~ 1 m. Although fringes in the phase contrast imaging locally appear in the near field, i.e. for an unfocused beam, they do not appear if radiation from an area larger than the coherence width is focused.

### Particle-in-cell simulation

**Figure 4:**
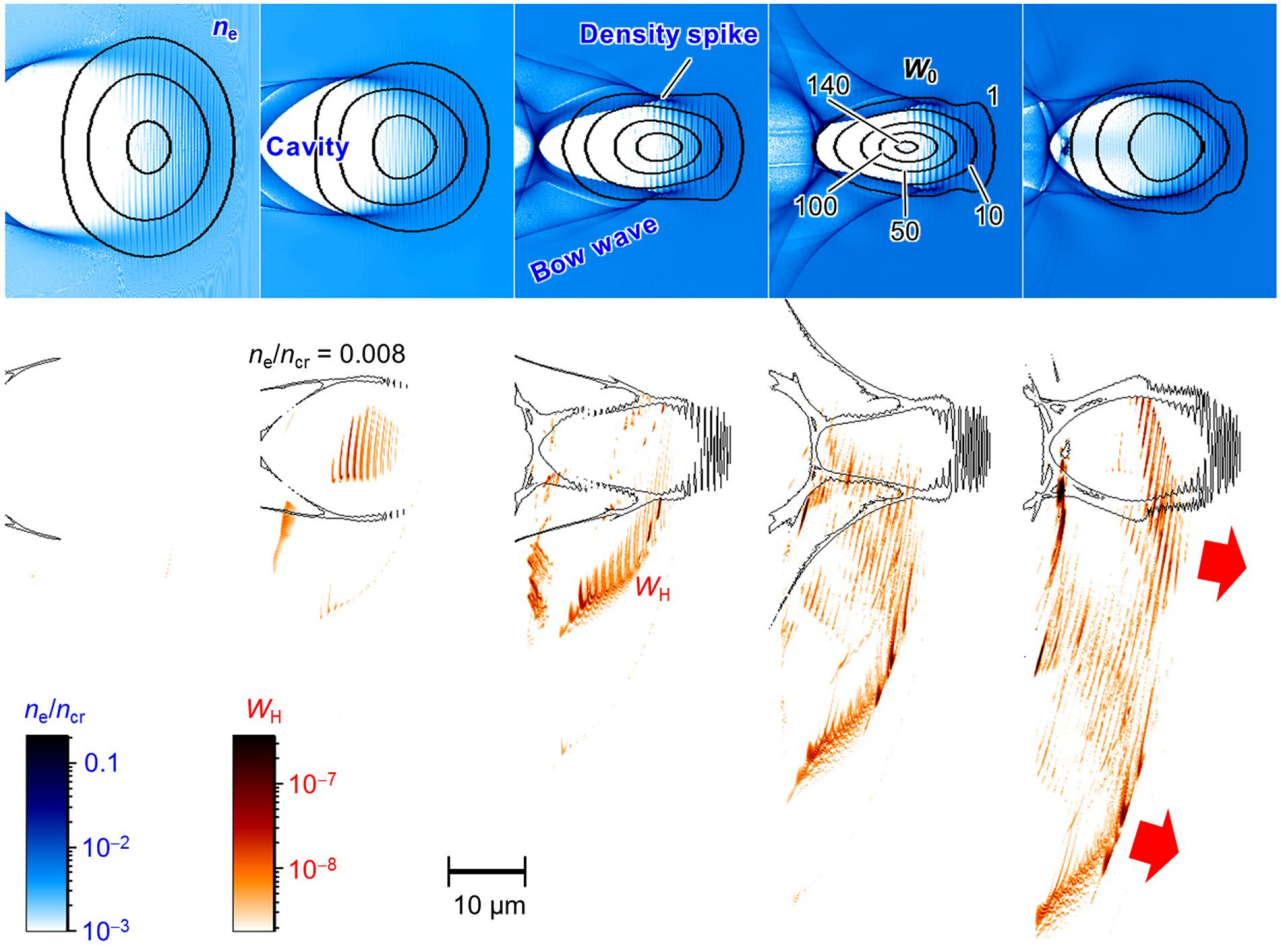
PIC simulation. Top: the laser pulse, represented by the curves of irradiance *W*_0_ in units of I_R_ = 2 × 10^18^ W/cm^2^, propagates in an inhomogeneous plasma indicated by the electron density *n*_e_ (blue, in units of *n*_cr_ = 1.7 × 10^21^ cm^−3^). Bottom: the irradiance *W*_H_ of the high-frequency electromagnetic field with photon energy from 60 to 90 eV emitted between the angles of 8° and 18° (corresponding to the acceptance angle in the experiment) with a superimposed electron density curve of *n*_e_/*n*_cr_ = 0.008. The time between panels is 270 fs. The rightmost panel corresponds to the centre of the jet.

We performed a particle-in-cell (PIC) simulation of an intense laser pulse propagating in plasma with parameters close to that of the experiment, Fig. 4, Supplementary Figures 6–10, and Supplementary Movies M1–M3.

In the simulation, the laser pulse propagated in plasma; its waist decreased while its irradiance increased due to relativistic self-focusing. The laser pulse pushed electrons aside (away from the laser axis), creating an almost void cavity in the electron density and an outgoing bow wave (top rows in Fig. 4 and Supplementary Figure 6). The electrostatic potential of the cavity pulled background electrons transversely toward the laser axis. When the cavity was closed so that electron flows propagated through each other, we observed the process called transverse wave breaking (TWB)^26^. The interpenetrating transverse flows of these electrons formed a characteristic outline (folding), denoted in Supplementary Figure 6 as TWB. At a later time, longitudinal wave breaking^25^ occurred, leading to the injection^35^ of an electron bunch into the cavity (the rightmost panel of the top row in Supplementary Figure 6).

The electron density spike was formed at the location, where the bow wave detached from the cavity wall, Fig. 4, the 2^nd^ top frame. The spike size was 14 nm Full Width at Half Maximum (FWHM), Supplementary Figure 7. For the peak density, the estimated particle spacing was ~1 nm, much smaller than the wavelength of the soft x-ray radiation observed in our experiments, fulfilling the necessary conditions for constructive interference. The spike moved forward entering the higher amplitude regions of the laser pulse, where it started to emit high-frequency radiation in the form of short pulse trains, Fig. 4, bottom frames.

The electromagnetic field of the laser pulse formed a density distribution modulated with the laser wavelength near the density spike, as seen in Fig. 4. At each moment of time the density distribution was renewed by electrons which were unperturbed before the laser pulse reached them. In the same fashion, the density peak at every moment of time consisted of different electrons, therefore its motion was determined by the change of the electron flow properties. Since the electron flow was modulated by the laser field, it was this field that ultimately determined the apparent motion of the density peak.

The electron motion in the laser field was substantially nonlinear because the laser intensity was high. The electron flow was modulated by the laser pulse in an oscillatory manner, with the period corresponding to the laser pulse carrier frequency. When the electron acceleration reached the maximum, its emission was the most intense. Correspondingly, the constructive interference of radiation from all such electrons flowing through the density spike produced a short (attosecond) high-frequency coherent electromagnetic pulse, in accordance with the BISER concept. Such pulses were emitted every laser wave period until the density peak left the high field region of the laser pulse, as seen in our simulations. Periodicity in the short pulse emission produced harmonics, the overall duration of which was determined by the density spike dynamics near the high field region and laser pulse evolution near the point of self-focusing.

With the change of the density spike motion direction, its emission direction accordingly changed. An x-ray searchlight formed in this way illuminating the detector within its acceptance angle at a certain time. Both of the electron spikes emitted high-frequency radiation in a relatively wide angle around the laser axis resulting in two point-like sources seen in the acceptance angle of the Imager and Spectrograph.

An electromagnetic field seen near the electron bunch injected^35^ into the cavity (Fig. 4, right; also in the rightmost panels of Supplementary Figure 6) mainly was not emitted, i.e. not converted into a travelling wave, because it was the field associated with the electric charge and current of the bunch^36^. In the bunch’s proper reference frame, this field was the electrostatic field of the electrons constituting the bunch plus some small low-frequency electromagnetic component due to relative motion of electrons in the bunch. Only the density spikes could produce high harmonics of the optical base frequency, because they emitted trains of short pulses, Fig. 2c.

Two density spikes produced soft x-ray emission in the form of trains of short pulses (Fig. 2c,d and Fig. 4, bottom row), carrying a total energy of 120 nJ within the photon energy range from 60 to 90 eV propagating into the acceptance angle of the detectors. The individual short pulses corresponding to the density spike oscillations had the duration of 170 attoseconds (Full Width at Half Maximum, FWHM), which was comparable with the bandwidth-limited 150 attosecond pulse estimated from the experimental spectrum within the same photon energy range, Fig. 2d.

## Discussion

Our model predicts that the two emitters situated on either side of the laser pulse should be equal for the case of emission lasting for several laser cycles or longer. Indeed, this was observed in the simulation, Supplementary Figures 6, 9, 10 and Movies 2 and 3. However, the two emitters seen by the Spectrograph (Fig. 2) and Imager (Fig. 3) were different in intensity (see also Supplementary Figures 2d and 2e). This was a consequence of our asymmetric detector setup, where both detectors were situated 13° off-axis (Supplementary Figures 2a and 3a), thus observing only part of the angular distribution. This difference was also seen in the simulation results showing only radiation propagating into the detector acceptance angle, as in Fig. 4, Supplementary Figure 8, and Movie M1. We note that an additional reason why the two emitters may differ could be a laser spot asymmetry. However, in our case this had a minor effect due to a rather symmetric spot, Supplementary Figure 1b.

The sub-micron size of the observed emitters makes them unique among x-ray sources, especially coherent ones. Other sources, such as coherent and incoherent x-rays due to atomic transitions^37^, recollision process^12^, betatron radiation^3,5^, and nonlinear Thomson scattering^2,4^, originate from regions with typical size from a few to several hundred micrometres, as determined by the laser focal spot, electron bunch oscillation amplitude, or plasma size. The fact that our pulse duration was close to the bandwidth limit gives the potential to achieve pulses as short as the present record of several tens of attoseconds^38^ demonstrated with the harmonics generated by the recollision process.

Our findings allow to estimate the power and brightness of the observed point-like x-ray sources. Assuming a 170 as x-ray pulse duration (shown by the simulation) and a pulse energy of 20 nJ within a single pulse of the train, we estimate the emitter pulse power as 0.1 GW. The estimated peak spectral brightness at a wavelength of 18 nm is 10^27^ photons/mm^2^ ·mrad^2^·s in 0.1% bandwidth, which is 5–6 orders of magnitude brighter than synchrotrons and 2–3 orders of magnitude lower than that provided by the brightest available soft x-ray sources, free-electron lasers, operating at similar wavelengths^39^; however, our source is much more compact.

The BISER concept, on the one hand paves the way towards sources of travelling waves based on singularities formed in media under controllable conditions and on the other hand allows the existence of singularities in multi-stream flows to be revealed. Both of these aims were achieved in our experiments and numerical simulations. Remarkably, the source size is orders of magnitude smaller than the driving agent. In our case, it was dramatically smaller than the laser pulse focus and even its smallest characteristic scale (laser wavelength). We note that the apparent source size and divergence of the coherent emission depend on the spectral range which is searched. A smaller source is seen at shorter wavelengths.

From a broader perspective, the BISER concept is applicable to all kinds of travelling waves. For example, gravitational wave^40,41^ generation, as proposed in ultrahigh-energy accelerators^42,43^, can be significantly enhanced by intentionally induced density singularities in the particle bunches, which in the future may result in laboratory-based gravitational wave emitters. In cosmic media, BISER can explain bright emission of electromagnetic and/or gravitational waves, which provides a new framework for interpreting a broad range of observations. In particular, in media exhibiting multi-stream flows, the density singularities moving with relativistic speed emit coherent electromagnetic and/or gravitational waves, which are much brighter than the surrounding incoherent emission. These BISER sources can be the progenitors of such electromagnetic pulses as gamma-ray bursts^44^ and fast radio bursts^45^, and can enrich the range of theoretically predicted waveforms used for signal searches^41^ in modern gravitational wave astronomy. Finally we note that the apparent characteristics of these emitters depend on their motion. Approaching a terrestrial observer at relativistic speeds, they appear to the observer as more intense, higher-frequency bursts.

## Methods

### Relativistic plasma

Electron dynamics are relativistic when the electron velocity, *v*, becomes close to the speed of light in vacuum, *c*, so that the electron’s Lorentz factor is substantially greater than unity, γ = (1−*v* ^2^/*c* ^2^)^−1/2^»1. The electron dynamics in the laser field become relativistic when the laser dimensionless amplitude is comparable to or greater than unity^24^. The dimensionless amplitude is defined as *a*_0_ = *eE*_0_/*m*_e_ *cω*_0_ = (*I*_0_ */I*_R_)^1/2^, where *I*_R_ = π*c* ^5^ *m*_e_^2^/2*e* ^2^λ_0_^2^ ≈ 1.37 × 10^18^ W/cm^2^ × (λ_0_[μm])^−2^ for a linearly polarized field. The dimensionless amplitude characterizes the importance of relativistic effects in laser-plasma interactions. A laser is said to be relativistically strong when *a*_0_ ≥ 1. Here *E*_0_, *I*_0_, λ_0_ and *ω*_0_ are the laser electric field, irradiance, wavelength and angular frequency, respectively; *e* and *m*_e_ are the electron charge and mass, respectively; λ_0_[μm] denotes the wavelength in micrometres. Note that in the caption of Fig. 4 the value of *I*_R_ = 2 × 10^18^ W/cm^2^ is specified for the laser wavelength of λ_0_ = 810 nm.

The plasma density is characterized by its initial electron density, *n*_e_, with respect to critical density, *n*_cr_ = *m*_e_ *ω*_0_^2^/4*πe* ^2^ ≈ 1.1 × 10^21^ cm^−3^/(λ_0_[μm])^2^. If *n*_e_≪*n*_cr_, the plasma is transparent to electromagnetic radiation (in the small amplitude limit)^20^; then the plasma is said to be underdense.

A laser beam undergoes relativistic self-focusing^46–48^ when its peak power, *P*_0_, exceeds the threshold of *P*_SF0_ = *P*_c_ (*n*_cr_/*n*_e_), where *P*_c_ = 2*m*_e_^2^ *c* ^5^/*e* ^2^ ≈ 0.017 TW, ref.^48^. In the stationary self-focusing regime^49^, the self-focused spot diameter and dimensionless amplitude can be estimated as *d*_0_ = λ_0_(*a*_0_ *n*_cr_/*n*_e_)^1/2^/π and *a*_0_ = (8π*P*_0_ *n*_e_/*P*_c_ *n*_cr_)^1/3^, respectively.

A relativistically strong laser pulse propagating in underdense plasma excites wake waves exemplified by a longitudinal Langmuir wave^27^.

A relativistically strong laser pulse produces a prominent bow wave when its focal spot is narrower than the threshold, *d* < *d*_BW_ = 2λ_0_(*a*_0_ *n*_cr_/*n*_e_)^1/2^/π, ref.^29^. This can be achieved by a tight focusing of the laser beam or via relativistic self-focusing.

### Laser

We used the multi-terawatt femtosecond J-KAREN laser^50^ with the central wavelength of λ_0_ ≈ 810 nm. The laser pulses were linearly polarized. The pulse energy was controllably varied from *ℇ*_L_ = 0.1 to 0.7 J. The soft x-ray emission from point-like sources was observed with laser pulse energies higher than 0.18 J. The temporal pulse shape, Supplementary Figure 1a, was measured with the self-referenced spectral interferometry^51^, resulting in the FWHM duration of about 30 fs and effective duration of τ_Eff_ ≈ 35 fs. Here τ_Eff_ = ∫*P*_n_(*t*)d*t* is the area under the normalized power curve *P*_n_(*t*), ref.^52^. The peak power *P*_0_ calculated as *P*_0_ = *ℇ*_L_/τ_Eff_ varied from ~3 to 20 TW. The pulses were focused with an f/9 off-axis parabolic mirror. The focal spot measured in vacuum, Supplementary Figure 1b, had the FWHM spot diameter *d*_VAC_ ≈ 10 μm with a Strehl ratio of about 0.3. The estimated irradiance in the absence of plasma varied from *I*_Vac_ ≈ 2 × 10^18^ W/cm^2^ at the smallest pulse energy to *I*_Vac_ ≈ 10^19^ W/cm^2^ at the highest one. Correspondingly, the dimensionless laser amplitude varied from *a*_0,Vac_ ≈ 0.9 to 2.2. The laser parameters fluctuated slightly day to day; the actual values were used in the data analysis. The laser pulse and spot shape were good enough to prevent filamentation development, as confirmed by the plasma channel image, Supplementary Figure 1c.

It was essential that the laser beam underwent relativistic self-focusing, because the laser peak power, *P*_0_, significantly exceeded the relativistic self-focusing threshold *P*_SF0_. This effect greatly enhanced the laser irradiance and, correspondingly, the laser dimensionless amplitude. The achieved peak intensity was estimated to be in the range from *I*_0_ ≈ 6 × 10^19^ to 2 × 10^20^ W/cm^2^ for the parameters of the experiment. This effect also facilitated the tightest focusing which satisfied the condition for the bow wave formation by the laser pulse. The self-focused spot diameter, *d*_0_, estimated according to the stationary self-focusing regime^49^, was safely two times narrower than the bow wave formation threshold, *d*_BW_.

### Gas jet

We used a 1-mm orifice diameter supersonic helium gas jet with the Mach number of 3.3. The gas density distribution was measured for several backing pressures using optical interferometry with Ar gas. The Ar gas had negligible cluster formation under our conditions (Hagena’s parameter^53^ values *Γ* ^*^ < 7 × 10^4^), and thus its atomic density was nearly identical to He with the accuracy of several percent^54^. The estimated peak density error was ≈ 30% determined by the interferometry noise and reconstruction process.

The peak electron density in the helium jet, controlled by the backing pressure, was varied from *n*_e_ ≈ 1.4 × 10^19^ to 6 × 10^19^ cm^−3^. The density was calculated as twice the atomic density because helium is fully ionized for the intensities used in the experiment^55^. This density was much lower than the critical density *n*_cr_≈ 1.7 × 10^21^ cm^−3^ for our laser’s central wavelength of λ_0_ ≈ 810 nm.

### Diagnostics setup

We sequentially used two detectors, a Spectrograph and an Imager, placed behind the gas jet at an angle of 13° with respect to the laser propagation. Both detectors used the same near-normal-incidence imaging mirror (schematically represented by “imaging optics” in Fig. 1b). The mirror had a 48 mm open diameter and a 500 mm radius of curvature. Due to the near-normal incidence, the mirror provided a large acceptance angle, collecting radiation propagating in a ± 5° cone around the angle of 13° with respect to the laser axis in the polarization plane (in other words, the cone was bounded by the angles from 8° to 18° in that plane). The mirror formed low-aberration magnified images of the source on the imaging sensors, either a back-illuminated Charge-Coupled Device (CCD) for the Spectrograph, Supplementary Figure 2, or a LiF crystal for the Imager, Supplementary Figure 3. As no single material can afford efficient normal-incidence reflection in the soft x-ray spectral region, we used an aperiodic Mo/Si multilayer coating^56^ designed for approximately uniform 11% reflectivity in the 12.4 to 20 nm spectral region^30^, corresponding to the photon energy range of 60 to 100 eV. To avoid detector exposure from laser and out-of-band plasma radiation, we used two free-standing optical blocking filters, specifically two 0.2 µm thick multilayer Zr/Al filters^31^ in the case of the Spectrograph and 0.1 µm Zr and 0.2 µm Zr/Al filters in the case of the Imager. A 0.4 T, 5 cm long magnet deflected charged particles away from the sensors.

### Spectrograph

This detector, Supplementary Figure 2, registered spatially-resolved spectra and spectrally-integrated images of electromagnetic radiation in the soft x-ray spectral region with the photon energies from 60 to 100 eV (corresponding to the wavelengths from 12.4 to 20 nm). The spectrograph throughput is shown in Supplementary Figure 4a. The sharp edge at 12.4 nm was due to the Silicon L absorption edge, while the roll-off at *λ* > 20 nm was due to the reduced filter transmission. A spectral resolution of about 0.1 nm was attained without a slit due to the small source size. The spatial resolution reached from several to 10 µm in the object space. Both spectral and spatial resolutions were determined by the 13.5 µm CCD pixel size and geometrical aberrations.

### Imager

This detector, Supplementary Figure 3, recorded spectrally-integrated images of soft x-ray plasma emission covering the same spectral region as the Spectrograph, from 60 to 100 eV, Supplementary Figure 4b. The Imager provided much higher, better than 1 μm, spatial resolution because we used a smaller incidence angle and a LiF crystal imaging sensor instead of the CCD. The high-frequency radiation caused formation of colour centres in the LiF crystal, recording the source images. These images were read out after the experiment with a fluorescence microscope^57^. Being excited by UV radiation the colour centres emitted visible light recorded by the microscope. The estimated depth of focus for the Imager was smaller than 10 μm, in the case of <1 μm objects. Therefore, the observation of two point-like sources indicated that the distance between them in the direction of observation was well below 10 μm; sources with larger longitudinal separation would appear blurred (defocused).

### Simulation

The simulation was done with the multi-parametric multi-dimensional code REMP, based on the Particle-in-Cell method^58^ and the density decomposition scheme^59^. The simulation configuration was two-dimensional. The simulation box size was 104λ_0_ and 180λ_0_ in the longitudinal and the transverse direction, respectively. Along those directions the mesh size was, respectively, λ_0_/400 and λ_0_/96; the time step was 2.426×10^−3^ λ_0_/*c*. The simulation was performed using the moving window technique^60^. In this technique, the simulation box (window) moves with the speed of light, *c*, in the longitudinal direction, in order to observe the laser pulse evolution and corresponding phenomena, neglecting processes far behind the laser pulse. The processes left behind the window boundaries could not influence the dynamics inside the window, because their influence propagated with the velocity not greater than the speed of light, *c*. The plasma was modelled by electrons on a background of immobile ions. In this approximation, the ion dynamics were neglected. Owing to large ion inertia, the ion response to the laser field and plasma wake fields was negligible on the timescale of the moving window. The maximum number of quasi-particles representing electrons was 2.2×10^9^.

In a previous publication^10^, we presented 3D simulations with lower resolution, where the 3D structure of the emitter can be seen. In the present work, the resolution is high enough to compensate numerical dispersion and to suppress the unphysical Cherenkov radiation in the spectral region of interest. For our simulation parameters, the maximum angle of the unphysical Cherenkov emission for particles moving in the laser propagation direction is below 5 degrees from the laser axis. Such radiation is not discernible even in Supplementary Figure 6, representing the radiation propagating into a cone spanning from −18 to +18 degrees, and certainly does not affect angles from 8 to 18 degrees and from −18 to −8 degrees.

The laser pulse had a Gaussian profile with FWHM duration of 38 fs and FWHM spot size of 13 μm with respect to irradiance. The laser pulse was linearly polarized in the transverse direction, in the plane of the simulation box. The shape of the laser pulse in terms of dimensionless amplitude was

1 $${A}_{0}(t,x,y)={a}_{0}{(\frac{1}{2})}^{{(2(x-t)/{L}_{x})}^{2}}(\frac{0.249}{0.25+{(y/{L}_{y})}^{2}}+4\times {10}^{-3}\exp (-2.77{(y/{L}_{y})}^{2})),$$

which was an approximation of the result of the laser pulse measurements. Here *L*_*x*_ = 20λ_0_, *L*_*y*_ = 25λ_0_.

The plasma density profile approximation was fitted from the gas jet measurements. In the transverse direction the plasma density was almost flat, while in the longitudinal direction it was given by the formula

2 $${n}_{{\rm{e}}}(x)={n}_{{\rm{e}},\max }(\frac{0.357}{1+3.41{(x/{S}_{x})}^{2}}+0.643\exp (-2.95{(x/{S}_{x})}^{2})),$$

where *S*_*x*_ = 525λ_0_.

Supplementary Figure 6 shows the laser pulse evolution (top panels, thick curves) in plasma (top panels, blue colour scale for electron density), and corresponding high-frequency emission going into the cone ±18° around the laser axis (bottom panels, orange-red colour scale). See also the Supplementary Figures 7–10 and Supplementary Movies M1–M3. In M1, the high-frequency emission going only into the experimental acceptance angle is shown. In M2, M3, the aperture is wider, from −18° to 18° around the laser axis. The Movie M3 presents the laser propagation in the global window, in contrast to M1 and M2 showing the processes in the moving window.

The high-frequency emission was generated in the electron density spikes, at the joint of the bow wave and the cavity wall, Supplementary Figure 7. Since the formation region of the high-frequency emission outgoing in the longitudinal direction has a minimum size of the order of the emission wavelength, it is sufficient to spatially resolve a reasonable range of wavelengths of the emission of interest. In the longitudinal direction, our simulations safely resolved the wavelengths down to λ_0_/67 = 12.15 nm, which corresponds to photon energies up to 102 eV. This covers the spectral range observed in the experiments. For the emission propagating in the transverse (perpendicular to the laser axis) direction, the resolved minimum wavelength was λ_0_/16 = 50.63 nm, corresponding to a maximum photon energy of 24.5 eV.

### Brightness estimate

We estimated the brightness of singular soft x-ray source using the measured spectrum (Supplementary Figure 5), effective angular width of ~5° measured with the Spectrograph in the intentionally defocused mode, calibrated with the shadow of parallel support structure (Supplementary Figure 2b), and time evolution from the PIC simulation (Fig. 2c). The source size in the laser polarization plane, 14 nm, was estimated from the simulation (Supplementary Figure 7), while in the perpendicular plane it was estimated from the experiment (Fig. 3).

## Electronic supplementary material

Supplementary information

Supplementary Movie M1

Supplementary Movie M2

Supplementary Movie M3

## References

[1] McPherson A (1987). Studies of multiphoton production of vacuum-ultraviolet radiation in the rare gases. J. Opt. Soc. Am. B.

[2] Esarey E, Ride SK, Sprangle P (1993). Nonlinear Thomson Scattering of Intense Laser-Pulses from Beams and Plasmas. Phys. Rev. E.

[3] Esarey E, Shadwick BA, Catravas P, Leemans WP (2002). Synchrotron radiation from electron beams in plasma-focusing channels. Phys. Rev. E.

[4] Ta Phuoc K (2003). X-ray radiation from nonlinear Thomson scattering of an intense femtosecond laser on relativistic electrons in a helium plasma. Phys. Rev. Lett..

[5] Rousse A (2004). Production of a keV x-ray beam from synchrotron radiation in relativistic laser-plasma interaction. Phys. Rev. Lett..

[6] Popmintchev T (2012). Bright Coherent Ultrahigh Harmonics in the keV X-ray Regime from Mid-Infrared Femtosecond Lasers. Science.

[7] Takahashi EJ, Lan P, Mücke OD, Nabekawa Y, Midorikawa K (2013). Attosecond nonlinear optics using gigawatt-scale isolated attosecond pulses. Nature Communications.

[8] Krausz F, Ivanov M (2009). Attosecond physics. Rev. Mod. Phys..

[9] Corde S (2013). Femtosecond x rays from laser-plasma accelerators. Rev. Mod. Phys..

[10] Pirozhkov AS (2012). Soft-X-Ray Harmonic Comb from Relativistic Electron Spikes. Phys. Rev. Lett..

[11] Pirozhkov AS (2014). High order harmonics from relativistic electron spikes. New J. Phys..

[12] Corkum PB (1993). Plasma perspective on strong field multiphoton ionization. Phys. Rev. Lett..

[13] Kugland NL (2012). Self-organized electromagnetic field structures in laser-produced counter-streaming plasmas. Nature Phys..

[14] Remington BA, Arnett D, Drake RP, Takabe H (1999). Modeling Astrophysical Phenomena in the Laboratory with Intense Lasers. Science.

[15] Gregori G (2012). Generation of scaled protogalactic seed magnetic fields in laser-produced shock waves. Nature.

[16] Albertazzi B (2014). Laboratory formation of a scaled protostellar jet by coaligned poloidal magnetic field. Science.

[17] Esirkepov TZ, Bulanov SV (2012). Fundamental physics and relativistic laboratory astrophysics with extreme power lasers. European Astronomical Society Publications Series.

[18] Arnold, V. I. *Catastrophe theory*. 3^rd^ edn, (Springer-Verlag, 1992).

[19] Poston, T. & Stewart, I. *Catastrophe theory and its applications*. (Dover Pubns, 1996).

[20] Jackson, J. D. *Classical Electrodynamics*. 3^rd^ edn, (John Willey & Sons, 1998).

[21] Einstein A (1905). On the electrodynamics of moving bodies. Annalen der Physik.

[22] Kando M (2007). Demonstration of laser-frequency upshift by electron-density modulations in a plasma wakefield. Phys. Rev. Lett..

[23] Leemans WP (2003). Observation of Terahertz Emission from a Laser-Plasma Accelerated Electron Bunch Crossing a Plasma-Vacuum Boundary. Phys. Rev. Lett..

[24] Mourou GA, Tajima T, Bulanov SV (2006). Optics in the relativistic regime. Rev. Mod. Phys..

[25] Dawson JM (1959). Nonlinear Electron Oscillations in a ColdPlasma. Physical Review.

[26] Bulanov SV, Pegoraro F, Pukhov AM, Sakharov AS (1997). Transverse-Wake Wave Breaking. Phys. Rev. Lett..

[27] Akhiezer A, Polovin R (1956). Theory of wave motion of an electron plasma. Sov. Phys. JETP.

[28] Matlis NH (2006). Snapshots of laser wakefields. Nature Phys..

[29] Esirkepov TZ, Kato Y, Bulanov SV (2008). Bow Wave from Ultraintense Electromagnetic Pulses in Plasmas. Phys. Rev. Lett..

[30] Levashov VE, Mednikov KN, Pirozhkov AS, Ragozin EN (2004). Interaction of laser plasmas with noble gases. Plas. Phys. Rep..

[31] Volodin BA (2010). Multilayer thin-film filters of extreme ultraviolet and soft X-ray spectral regions. Bull. Rus. Ac. Sci.: Phys..

[32] Schnopper HW (1977). Diffraction grating transmission efficiencies for XUV and soft x rays. Appl. Opt..

[33] Henke, B. L., Gullikson, E. M. & Davis, J. C. X-Ray Interactions: Photoabsorption, Scattering, Transmission, and Reflection at E = 50–30,000 eV, Z = 1–92. *At*. *Data Nucl*. *Data Tables* **54**, 181–342, 10.1006/adnd.1993.1013 (1993), available online at http://henke.lbl.gov/optical_constants/.

[34] Mandel, L. & Wolf, E. *Optical coherence and quantum optics*. (Cambridge University Press, 1995).

[35] Tajima T, Dawson J (1979). Laser electron accelerator. Phys. Rev. Lett..

[36] Liseikina TV, Califano F, Vshivkov VA, Pegoraro F, Bulanov SV (1999). Small-scale electron density and magnetic-field structures in the wake of an ultraintense laser pulse. Phys. Rev. E.

[37] Janev, R. K., Presnyakov, L. P. & Shevelko, V. P. *Physics of highly charged ions*. 2 edn, Vol. 13 (Springer-Verlag, 2012).

[38] Zhao K (2012). Tailoring a 67 attosecond pulse through advantageous phase-mismatch. Opt. Lett..

[39] Ackermann W (2007). Operation of a free-electron laser from the extreme ultraviolet to the water window. Nat. Photon.

[40] Einstein A, Rosen N (1937). On gravitational waves. Journal of the Franklin Institute.

[41] Abbott BP (2016). Observation of Gravitational Waves from a Binary Black Hole Merger. Phys. Rev. Lett..

[42] Diambrini Palazzi G, Fargion D (1987). On gravitational radiation emitted by circulating particles in high energy accelerators. Physics Letters B.

[43] Nikishov AI, Ritus VI (2010). Gravitational radiation of systems and the role of their force field. Physics-Uspekhi.

[44] Gehrels N, Mészáros P (2012). Gamma-Ray Bursts. Science.

[45] Spitler LG (2016). A repeating fast radio burst. Nature.

[46] Askaryan GA (1962). Effect of the gradient of a strong electromagnetic ray on electrons and atoms. Zhur. Eksptl’. i Teoret. Fiz..

[47] Litvak A (1970). Finite-amplitude wave beams in a magnetoactive plasma. Sov. Phys. JETP..

[48] Sun G-Z, Ott E, Lee YC, Guzdar P (1987). Self-focusing of short intense pulses in plasmas. Phys. Fluids.

[49] Bulanov SS (2010). Generation of GeV protons from 1 PW laser interaction with near critical density targets. Phys. Plasmas.

[50] Kiriyama H (2012). Temporal contrast enhancement of petawatt-class laser pulses. Opt. Lett..

[51] Moulet A, Grabielle S, Cornaggia C, Forget N, Oksenhendler T (2010). Single-shot, high-dynamic-range measurement of sub-15 fs pulses by self-referenced spectral interferometry. Opt. Lett..

[52] Pirozhkov AS (2017). Approaching the diffraction-limited, bandwidth-limited Petawatt. Opt. Express.

[53] Hagena OF (1992). Cluster ion sources. Rev. Sci. Instr..

[54] Boldarev AS, Gasilov VA, Faenov AY, Fukuda Y, Yamakawa K (2006). Gas-cluster targets for femtosecond laser interaction: Modeling and optimization. Rev. Sci. Instr..

[55] Popov VS (2004). Tunnel and multiphoton ionization of atoms and ions in a strong laser field (Keldysh theory). Physics-Uspekhi.

[56] Pirozhkov AS, Ragozin EN (2015). Aperiodic multilayer structures in soft X-ray optics. Phys. Usp..

[57] Faenov AY (2009). Submicrometer-resolution *in situ* imaging of the focus pattern of a soft x-ray laser by color center formation in LiF crystal. Opt. Lett..

[58] Hockney, R. W. & Eastwood, J. W. *Computer Simulation Using Particles*. (McGraw-Hill, 1981).

[59] Esirkepov TZ (2001). Exact charge conservation scheme for Particle-in-Cell simulation with an arbitrary form-factor. Comput. Phys. Comm..

[60] Fidel B, Heyman E, Kastner R, Ziolkowski RW (1997). Hybrid Ray–FDTD Moving Window Approach to Pulse Propagation. J. Comput. Phys..

