# Supplementary material for: Burst intensification by singularity emitting radiation in multi-stream flows
Source: Sci Rep. 2017 Dec 21;7:17968. doi: 10.1038/s41598-017-17498-5 (PMC5740116; doi:10.1038/s41598-017-17498-5)
Supplement: Supplementary file 1 — Supplementary information [file 41598_2017_17498_MOESM1_ESM.pdf]

Burst intensification by singularity emitting radiation in multi-stream flows

A. S. Pirozhkov[1*], T. Zh. Esirkepov[1*], T. A. Pikuz[2,3], A. Ya. Faenov[3,4], K. Ogura[1], Y. Hayashi[1], H. Kotaki[1], E. N. Ragozin[5,6], D. Neely[7,8], H. Kiriyama[1], J. K. Koga[1], Y. Fukuda[1], A. Sagisaka[1], M. Nishikino[1], T. Imazono[1], N. Hasegawa[1], T. Kawachi[1], P. R. Bolton[1,13], H. Daido[9], Y. Kato[10], K. Kondo[1], S. V. Bulanov[1,11,12], and M. Kando[1]

Supplementary Information

[1] Kansai Photon Science Institute, National Institutes for Quantum and Radiological Science and Technology, 8-1-7 Umemidai, Kizugawa-city, Kyoto 619-0215, Japan. [2]Graduate School of Engineering, Osaka University, 2-1 Yamadaoka, Suita, Osaka 565-0871, Japan. [3]Joint Institute for High Temperatures of the Russian Academy of Sciences, Izhorskaja Street 13/19, Moscow 127412, Russia. [4]Open and Transdisciplinary Research Initiatives, Osaka University, Suita, Osaka, 565-0871, Japan. [5]P. N. Lebedev Physical Institute of the Russian Academy of Sciences, Leninsky Prospekt 53, Moscow 119991, Russia. [6]Moscow Institute of Physics and Technology (State University), Institutskii pereulok 9, Dolgoprudnyi, Moscow Region 141700, Russia. [7]Central Laser Facility, Rutherford Appleton Laboratory, STFC, Chilton, Didcot, Oxon OX11 0QX, UK. [8]Department of Physics, SUPA, University of Strathclyde, Glasgow G4 0NG, UK. [9] Naraha Remote Technology Development Center, Japan Atomic Energy Agency, Naraha-machi, Fukushima 979-0513, Japan. [10]The Graduate School for the Creation of New Photonics Industries, 1955-1 Kurematsu-cho, Nishiku, Hamamatsu, Shizuoka 431-1202, Japan. [11]A. M. Prokhorov Institute of General Physics of the Russian Academy of Sciences, Vavilov Street 38, Moscow 119991, Russia. [12] Institute of Physics of the Czech Academy of Sciences, v.v.i. (FZU), ELI-Beamlines Project, Na Slovance 1999/2, 182 21 Prague, Czech Republic. [13]Present address: Chair of Experimental Physics and Medical Physics, Faculty of Physics, Ludwig-Maximilians-Universität München, Am Coulombwall 1, D-85748 Garching b. München, Germany.

Supplementary Figures

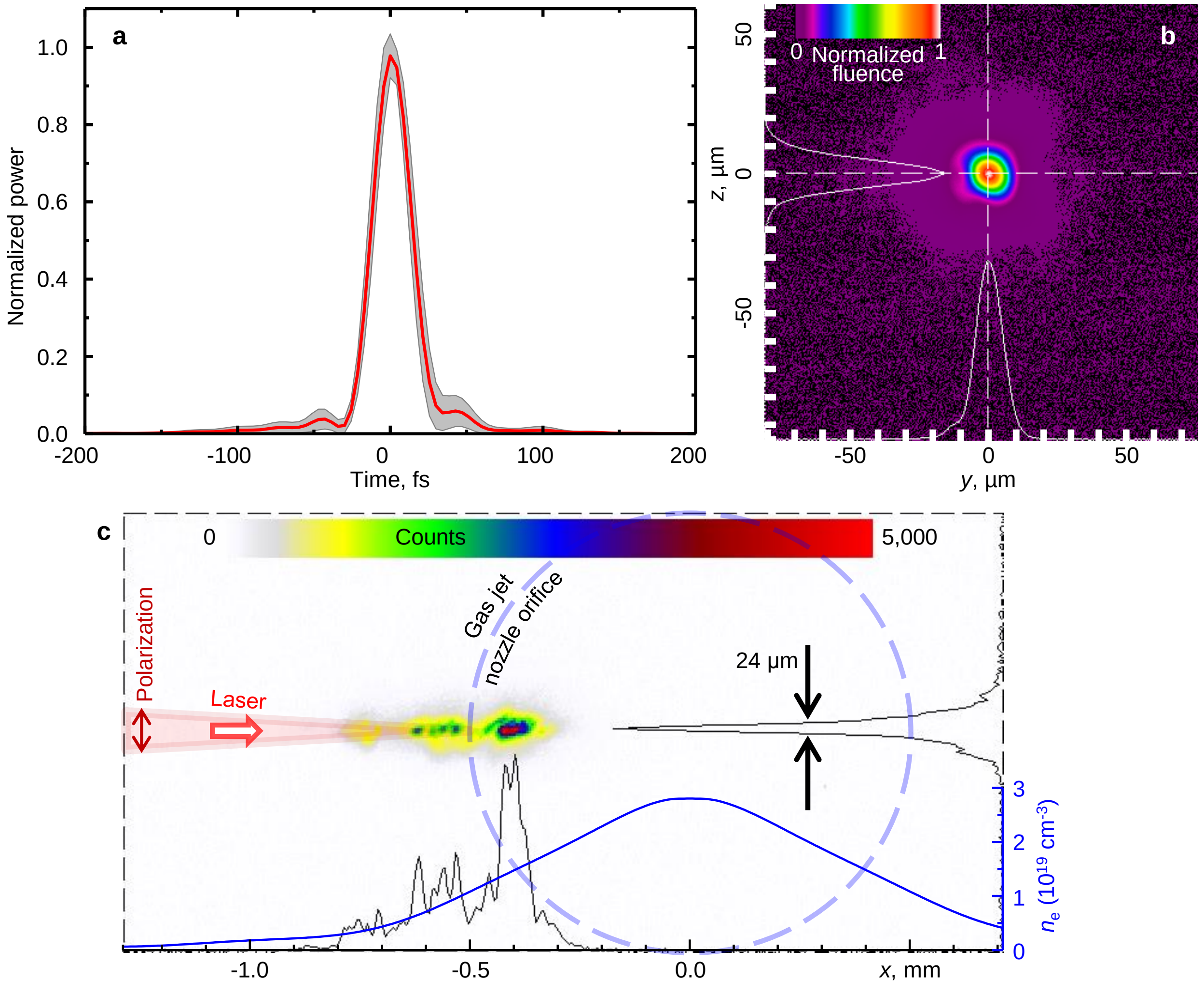

Supplementary Figure 1 | Laser pulse properties. (**a**) Pulse shape measured with self-referenced spectral interferometry[51]; the thick red line shows the average and the grey area depicts the standard deviation over 113 single shots. (**b**) Focal spot shape, the magnified image on a Charge Coupled Device (CCD) obtained with an achromatic doublet lens. (**c**) The laser pulse irradiating the gas jet formed a plasma channel image (yellow-green-blue-red colour scale) which displays no signs of filamentation. The dark-red arrow indicates the laser polarization direction.

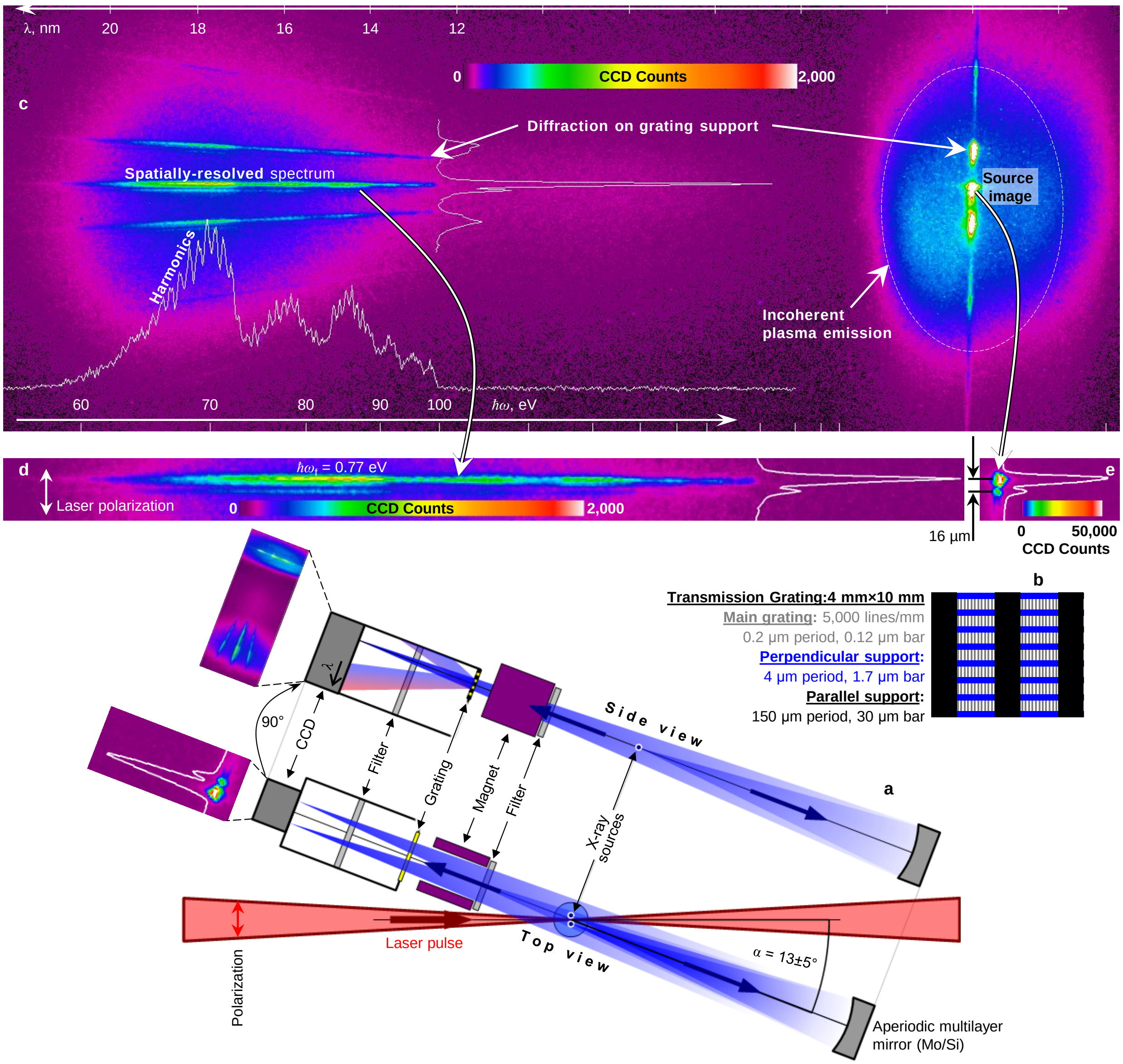

Supplementary Figure 2 | Spectrograph and its data. (**a**) Experimental setup schematic of the Spectrograph (not to scale). The laser pulse irradiated the gas jet producing x-ray emission. The red arrow denotes the laser polarization direction. The spherical mirror with aperiodic Mo/Si multilayer coating, set at the incidence angle of 1.8°, imaged the soft x-ray source to the back-illuminated Charge-Coupled Device (CCD) with the magnification of M = 6.85. Two Zr/Al optical blocking filters, 0.2 μm thick, rejected laser and out-of-band plasma

radiation. The magnet deflected charged particles. The transmission diffraction grating was used to obtain spectrally-resolved source images. In addition to the 5000 lines/mm main grating, there was also a 250 lines/mm periodic support mesh, (**b**), which caused diffraction in the perpendicular direction, helping spectral identification. The parallel support mesh with the period of 150 μm was used for angular scale calibration in an intentionally defocused mode. An example of the raw data is shown in (**c**); it corresponds to the shot shown in Figure 2a. The main grating formed the spectral decomposition in the horizontal direction, while the vertical direction represents the diffraction on the support mesh perpendicular to the main grating. Two sources of soft x-rays are seen in the first (horizontal) diffraction order, (**d**), and in the zeroth spectral order, (**e**) [note the dynamic range difference between the panels (d) and (e)]. The dashed ellipse in **c** denotes a dim incoherent plasma emission from area spanning about 500 μm. The spectrum lineout in (c), corresponding to the highest signal in the first (horizontal) diffraction order, exhibits two kinds of oscillations: slow ones being determined by the spectrograph throughput, Supplementary Figure 4a, and higher-frequency ones corresponding to the high-order harmonics of the optical base frequency (seen also in Figure 2a,b).

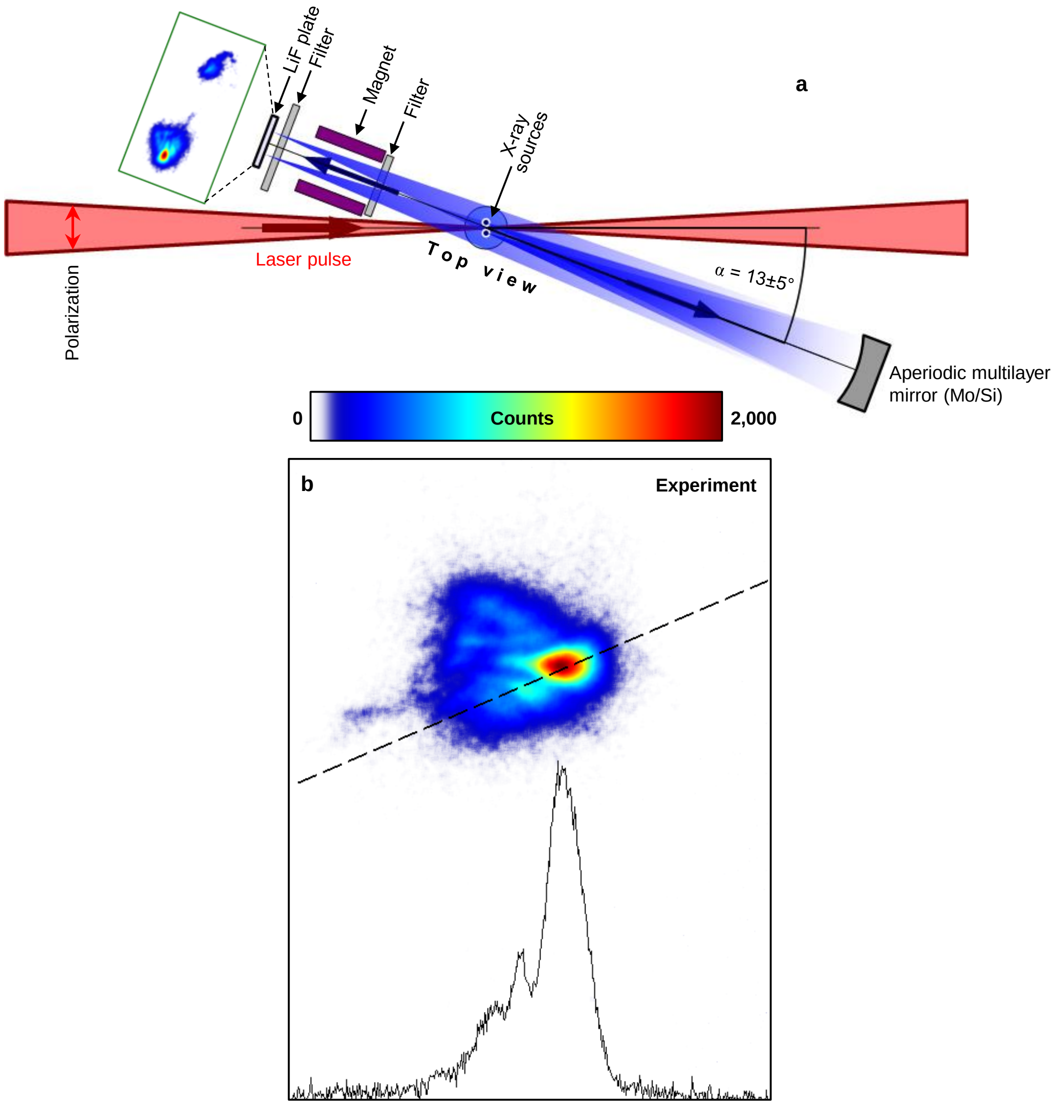

Supplementary Figure 3 | Imager and its data. Experimental setup schematic of the Imager (not to scale), (**a**). The spherical mirror with aperiodic Mo/Si multilayer coating, set at the near-normal incidence angle of 1.04°, imaged the soft x-ray source onto a high-resolution LiF crystal imaging sensor with the magnification of M = 5.53. The optical blocking filters were 0.1 µm Zr and 0.2 µm Zr/Al. The example of the data is shown in Figure 3. Its stronger emitter image is shown in panel (**b**); the lineout along the dashed line reveals fringes.

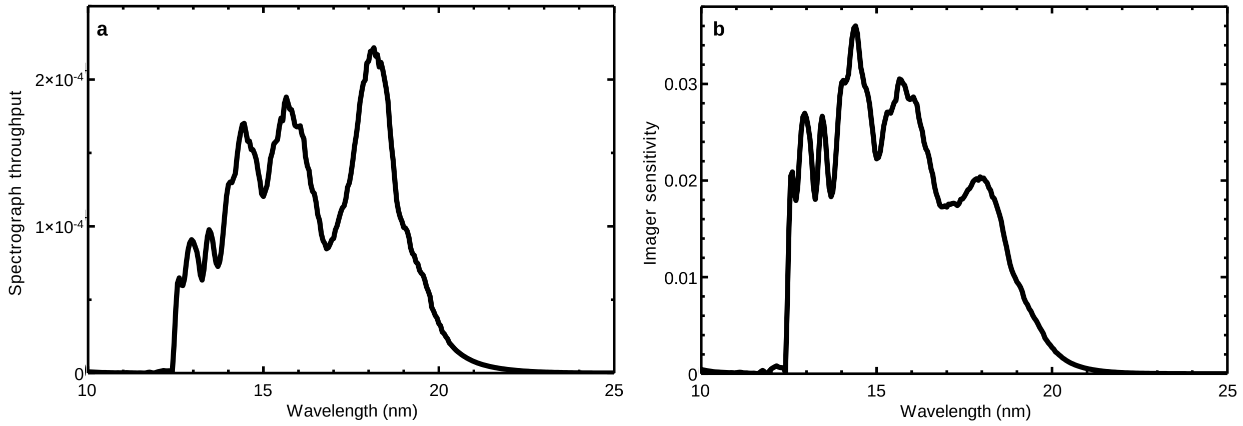

Supplementary Figure 4 | Spectral ranges of the Spectrograph and Imager. (**a**) The Spectrograph throughput, i.e. the product of the Mo/Si multilayer mirror reflectivity, transmission of two Zr/Al filters, grating efficiency, and CCD quantum efficiency. (**b**) The Imager spectral sensitivity curve, i.e. the product of the Mo/Si multilayer mirror reflectivity and transmission of Zr and Zr/Al filters.

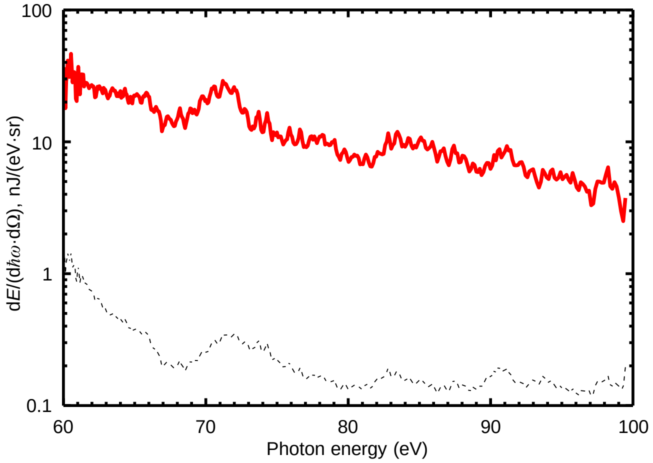

Supplementary Figure 5 | Singular emitter spectrum in the absolute units. The spectrum corresponds to the same shot as shown in Figure 2a and Supplementary Figure 2c-e. The dotted line represents the noise level.

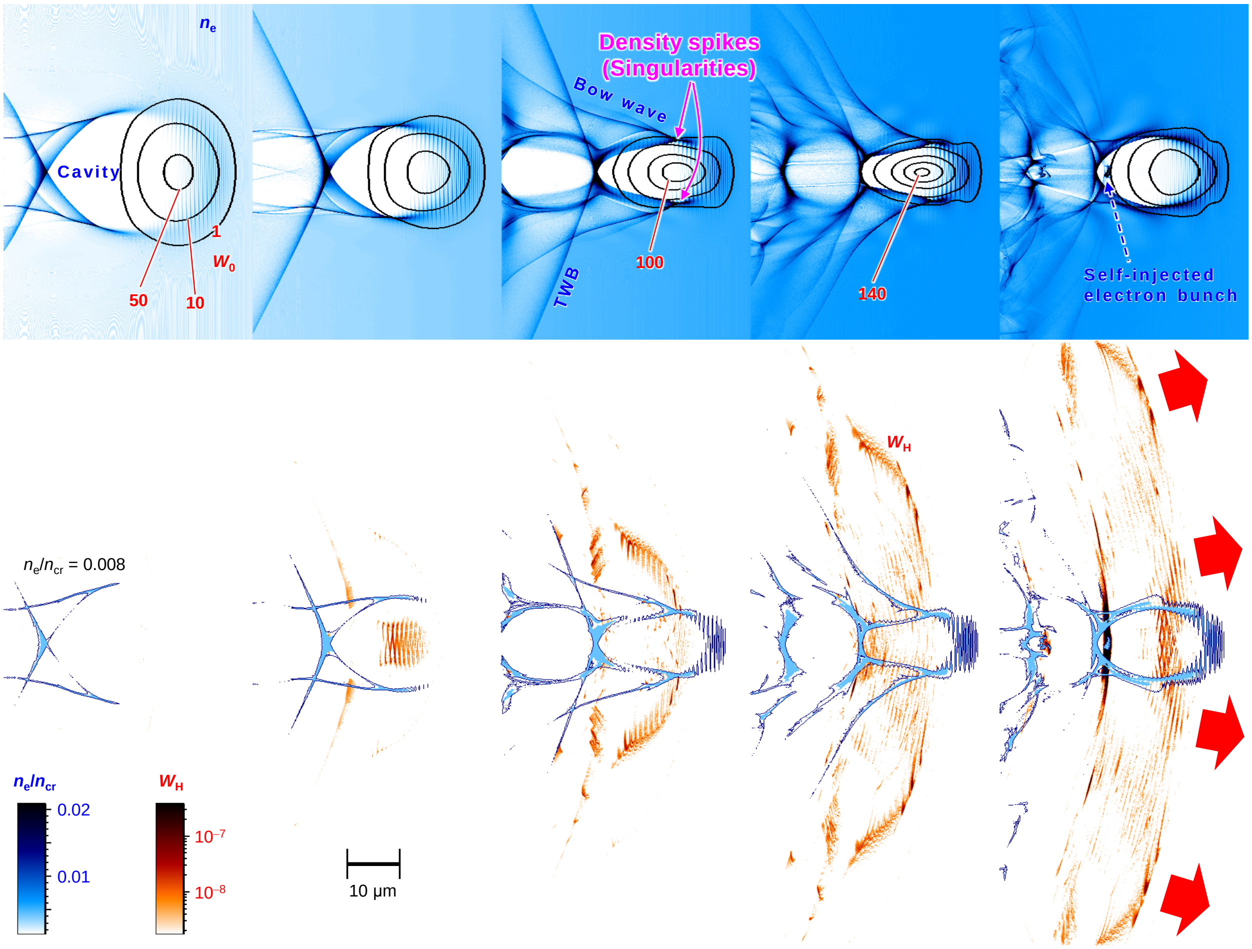

Supplementary Figure 6 | PIC simulation: the laser pulse evolution and the BISER. The panels show the same quantities and time moments as in Figure 4. Here the radiation propagating into a cone spanning from –18° to +18° is presented, in contrast to Figure 4, where only radiation propagating into the acceptance angle of the Imager and Spectrograph is shown, i.e. from 8° to 18° on one side of the laser axis. The scale of the electron density in the top row is linear rather than logarithmic as in Figure 4. TWB in the middle top panel stands for the Transverse Wave Breaking[26].

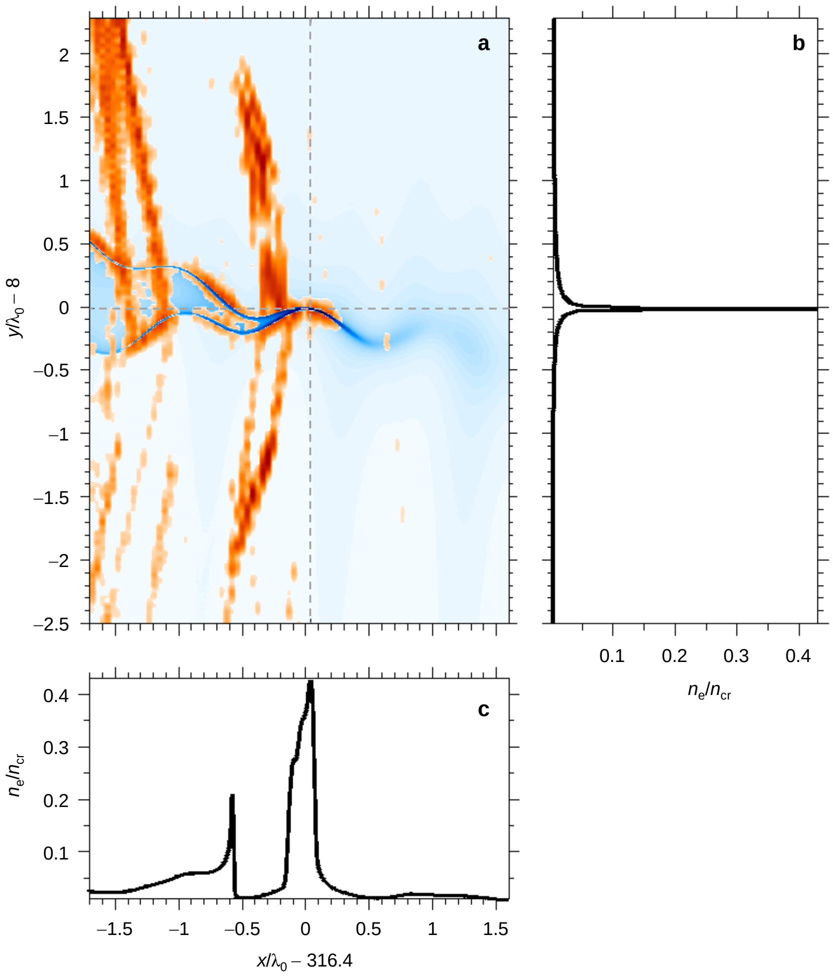

Supplementary Figure 7 | PIC simulation: close-up of the electron density spike. The region shown corresponds to the upper spike of the middle panel of Supplementary Figure 6. (**a**) Blue: electron density, red: high-frequency electromagnetic field energy density. (**b**), (**c**) electron density along corresponding dashed lines in frame (a).

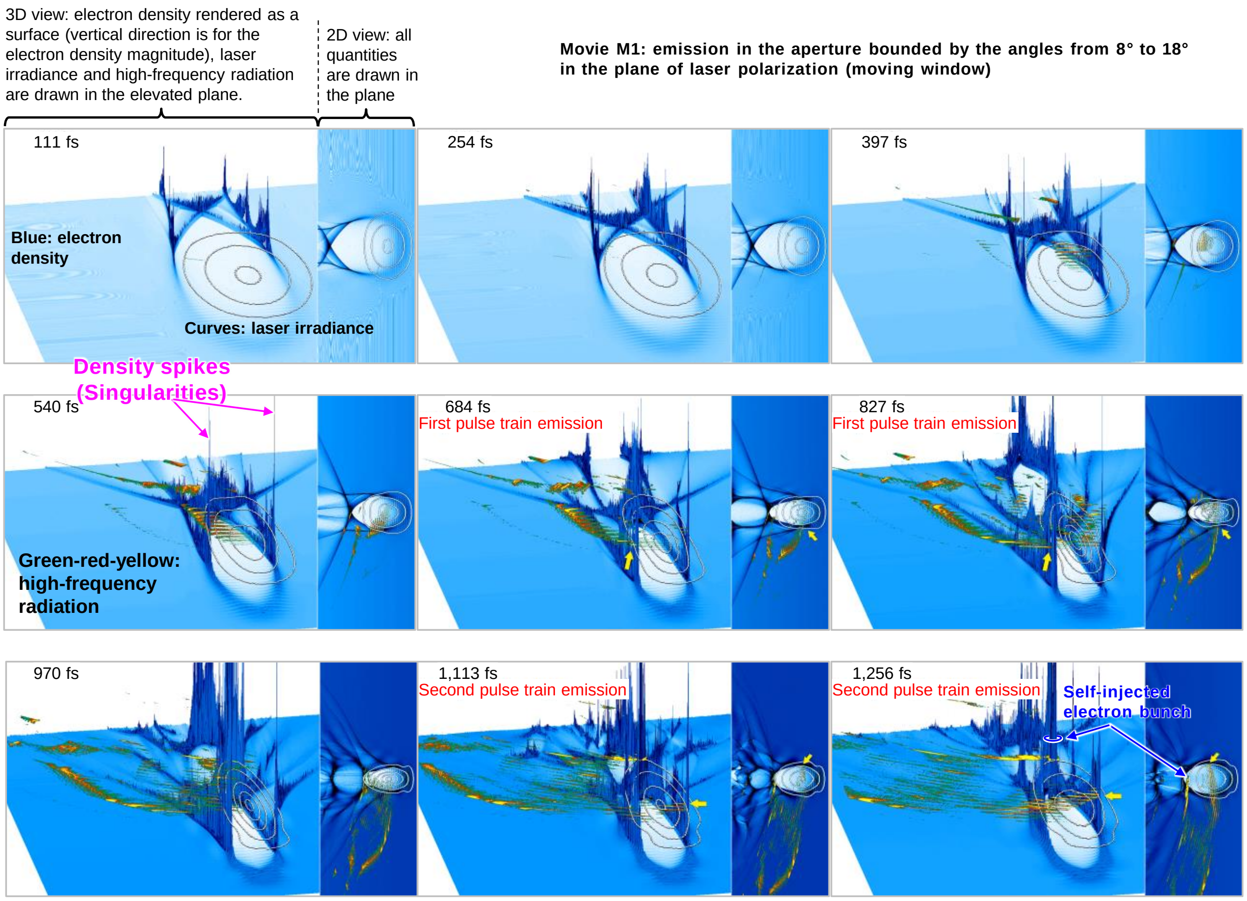

Supplementary Figure 8 | Selected frames from Supplementary Movie M1.

Movie M2: emission in the aperture bounded by the angles from -18° to 18° in the plane of laser polarization (moving window)

3D view: electron density rendered as a surface (vertical direction is for the electron density magnitude), laser irradiance and high-frequency radiation are drawn in the elevated plane.

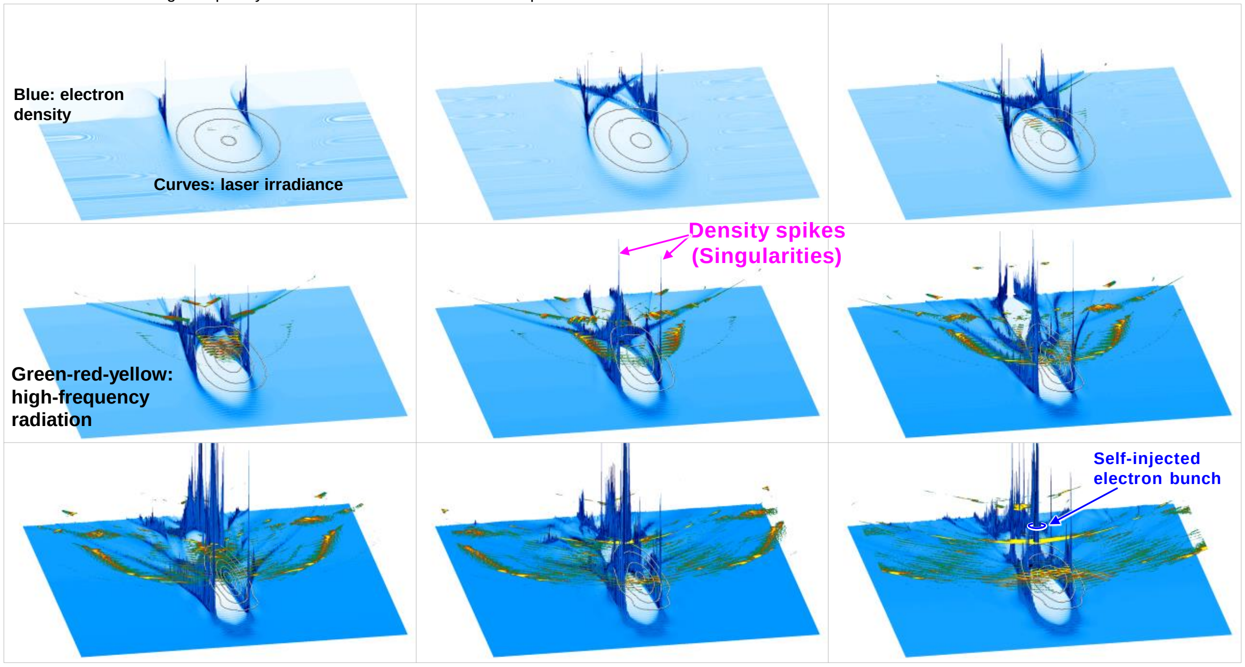

Supplementary Figure 9 | Selected frames from Supplementary Movie M2.

Movie M3: emission in the aperture bounded by the angles from -18° to 18° in the plane of laser polarization(global window)

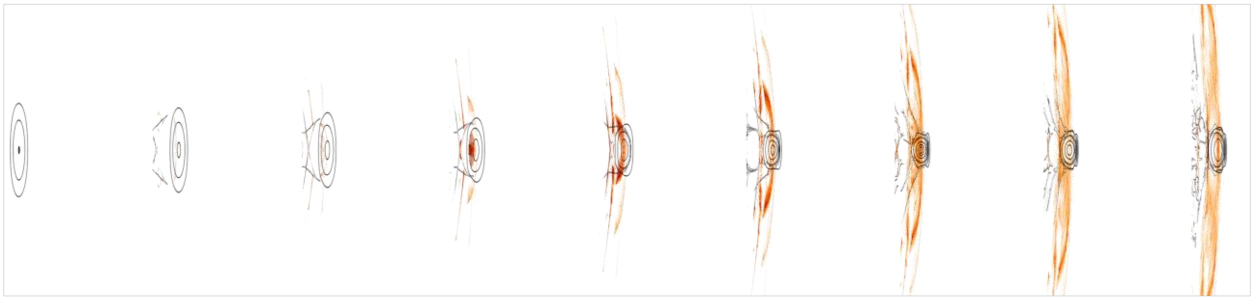

Supplementary Figure 10 | Selected frames from Supplementary Movie M3.

Supplementary Movies

Supplementary Movie M1. PIC simulation: the laser pulse evolution in the plasma and the BISER going into the experimental acceptance angle. High-frequency emission in the aperture bounded by the angles from 8° to 18° in the plane of laser polarization, in the window moving with the speed of light along the laser axis. The blue surface represents the electron density. Thick white curves show the laser irradiance levels of W_0 = 1, 10, 50, 100, 140, in units of I_R=2×10^{18} W/cm^2, as in Figure 4. The green-red-yellow colour scale reveals the high-frequency emission with photon energy from 60 to 90 eV; the two strongest emission moments are denoted by the yellow arrows and signs "First pulse train emission" and "Second pulse train emission". Although the emission of each train continues for a few hundred femtoseconds, the full duration of the resulting attosecond pulse train is several femtoseconds, because the emitter itself moves with the relativistic velocity, i.e. almost catching up with the outgoing radiation. We also note that at the presented (down-sampled) resolution, the pulses of high-frequency emission are invisible until they sufficiently diverge. When their intensity distribution starts to occupy more than several pixels, they become visible. This explains why the apparent intensity of short pulses sometimes increases with time (larger area looks more intense than the smaller one, especially near saturation).

Supplementary Movie M2. PIC simulation: the laser pulse evolution in plasma and the BISER going into angles from –18° to 18° near the axis. Curves and colour scales are the same as in **Supplementary Movie M1**.

Supplementary Movie M3. PIC simulation: the emission from the moving singularity. Each frame corresponding to the moving window, obtained in the simulation, appears in its right place in the global window. In order to ease the observation, the aspect ratio is set to 1/3 = horizontal/vertical. Thin curves correspond to the electron density constant value of n_e=0.008n_{cr}, where n_{cr}=1.7×10^{21} cm^{-3}. Thick oval-like curves show the laser irradiance levels of W_0 = 1, 10, 50, 100, 140, in units of I_R=2×10^{18} W/cm^2. The orange-red colour scale shows the high-frequency emission with photon energy within the 60 to 90 eV spectral range propagating into the angles from −18° to 18° with respect to the laser axis in the plane of laser polarization.
